# The potential role of temperate Japanese regions as refugia for the coral *Acropora hyacinthus* in the face of climate change

**DOI:** 10.1038/s41598-018-38333-5

**Published:** 2019-02-13

**Authors:** Aki Nakabayashi, Takehisa Yamakita, Takashi Nakamura, Hiroaki Aizawa, Yuko F Kitano, Akira Iguchi, Hiroya Yamano, Satoshi Nagai, Sylvain Agostini, Kosuke M. Teshima, Nina Yasuda

**Affiliations:** 1 Department of Marine Biology and Environmental Sciences, University of Miyazaki, Faculty of Agriculture, Gakuen- kibanadai-nishi-1-1, Miyazaki, 889-2192 Japan; 2 R&D Center for Submarine Resource, Japan Agency for Marine-Earth Science and Technology, 2-15, Natsushima-cho, Yokosuka-city, Kanagawa 237-0061 Japan; 3 Department of Transdisciplinary Science and Engineering, School of Environment and Society, Tokyo Institute of Technology, O-okayama 2-12-1-W8-5, Meguro-ku, Tokyo 152-8552 Japan; 4 Organization for Promotion of Tenure Track, University of Miyazaki, Faculty of Agriculture, Gakuen- kibanadai-nishi-1-1, Miyazaki, 889-2192 Japan; 5 Iriomote station, Tropical Biosphere Research Center, University of Ryukyus, 870 Uehara, Taketomi, Okinawa 907-1541 Japan; 6 National Institute for Environmental Studies, 16-2 Onogawa, Tsukuba-shi, Ibaraki 305-8506 Japan; 7 Department of Bioresources Engineering, National Institute of Technology, Okinawa College, 905 Henoko, Nago-City, Okinawa 905-2192 Japan; 8 Institute of Geology and Geoinformation, National Institute of Advanced Industrial Science and Technology (AIST), 1-1-1 Higashi, Tsukuba, Ibaraki 305-8567 Japan; 9 Research Center for Aquatic Genomics, National Research Institute of Fisheries Science, 2-12-4 Fukuura, Kanazawa-ku, Yokohama, Kanagawa 236-8648 Japan; 10 Shimoda Marine Research Center, University of Tsukuba, Shimoda 5-10-1, Shizuoka, 415-0025 Japan; 11 Department of Biology, Faculty of Science, Kyushu University 744 Motooka, Nishi-ku, Fukuoka, 819-0395 Japan

## Abstract

As corals in tropical regions are threatened by increasing water temperatures, poleward range expansion of reef-building corals has been observed, and temperate regions are expected to serve as refugia in the face of climate change. To elucidate the important indicators of the sustainability of coral populations, we examined the genetic diversity and connectivity of the common reef-building coral *Acropora hyacinthus* along the Kuroshio Current, including recently expanded (<50 years) populations. Among the three cryptic lineages found, only one was distributed in temperate regions, which could indicate the presence of Kuroshio-associated larval dispersal barriers between temperate and subtropical regions, as shown by oceanographic simulations as well as differences in environmental factors. The level of genetic diversity gradually decreased towards the edge of the species distribution. This study provides an example of the reduced genetic diversity in recently expanded marginal populations, thus indicating the possible vulnerability of these populations to environmental changes. This finding underpins the importance of assessing the genetic diversity of newly colonized populations associated with climate change for conservation purposes. In addition, this study highlights the importance of pre-existing temperate regions as coral refugia, which has been rather underappreciated in local coastal management.

## Introduction

Coral reef ecosystems harbour high biodiversity, supporting almost 30% of marine coastal species^1^. However, reef-building corals have a relatively narrow range of temperature tolerance and are considered especially vulnerable to climate change. The most well-known impact of warming oceans on corals is the increase in mass coral bleaching^2^. In addition, climate change exacerbates local stresses by reducing water quality, which further threatens coral reefs^2^. In contrast to tropical and subtropical regions where coral abundance is declining due to increasing water temperatures, rapid poleward expansions of the ranges of hermatypic corals have been reported in temperate Japanese regions^3^ and the Mediterranean Sea^4^. Given that all of the range-expanding coral species are classified as Near Threatened or Vulnerable on the IUCN Red List, temperate regions may serve as refugia for hermatypic coral species in the near future^3^ if the coral species can overcome the seasonal fluctuations in temperature and reduced light intensities^5^. Among the multiple definitions of refugia, we use the term as defined by Keppel and Wardell-Johnson^6^. In this paper, authors noted that “Refugia are habitats that components of biodiversity retreat to, persist in and can potentially expand from under changing environmental conditions and facilitates the persistence of species under anthropogenic climate change.” Population connectivity and relatively high genetic diversity are crucial for a population to expand and persist under climate change^7^; thus, these characteristics are important for the populations in potential refugia.

Winter water temperature has risen by 1.45 °C over the last 100 years in the temperate Kuroshio Current region in Japan (http://www.data.jma.go.jp/gmd/kaiyou/data/shindan/a_1/japan_warm/japan_warm.html), causing coral expansion and producing potential coral refugia at the edges of their distribution ranges^3^. While such temperate region can be potential coral refugia, temperate peripheral populations may suffer from reduced genetic diversity due to small population sizes and isolation^8^. However, no studies have assessed the genetic diversity or structures of such coral populations, including the recently colonized temperate populations that expanded within the last 50 years as a result of climate change along the Kuroshio Current. In addition, there is a lack of knowledge of the oceanographic larval dispersal processes that shape the genetic variations and population structures of corals in the high-latitude regions of Japan.

This study aimed to assess the genetic structures of recently expanded populations and examine the potential role of temperate regions as coral refugia in Japan. We thus examined the genetic diversity and meta-population structures of the subtropical to northernmost temperate populations of the reef-building coral *Acropora hyacinthus*, and the results were further compared with contemporary larval dispersal simulations in an oceanographic model.

**Figure 1:**
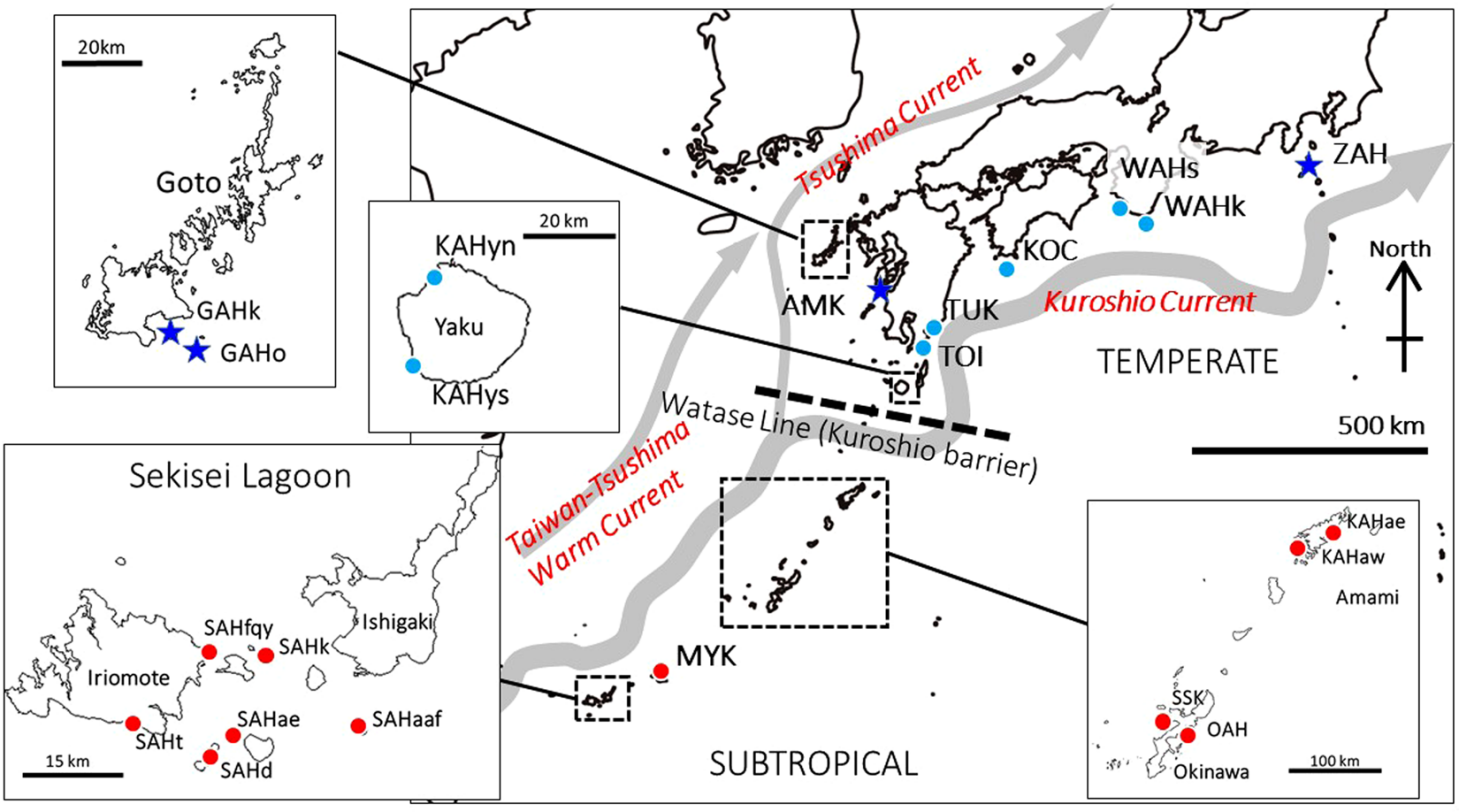
Sampling sites in this study. The thickest grey curve indicates the Kuroshio Current. The circles represent pre-existing populations, and the stars indicate recently expanded populations.

Our target species, *Acropora hyacinthus* (Dana, 1846), is widely distributed in the tropical, subtropical, and some part of temperate regions of the Indo-Pacific^9^. Northward range expansion of *A*. *hyacinthus* has been observed in the temperate regions of Japan for the last 50 years^3^. Previous studies that included some temperate populations found cryptic lineages (species) of *A*. *hyacinthus*^10,11^. The *A*. *hyacinthus* species complex is a hermaphroditic, broadcast-spawning species whose fertilization takes place in the water column. More than 70% of the larvae settle within one week^12^ but can survive in the water column for up to 5–6 weeks^13^. *A*. *hyacinthus* is categorized as Near Threatened on the IUCN red list. *A*. *hyacinthus* is one of the key reef-building species on tropical reefs^14^ and can represent as much as 36% of the coral coverage in some high-latitude areas^15^. These ecological features of this species are suitable for comparing the genetic diversity and connectivity of populations among temperate and subtropical regions. We defined the region south of the Watase Line^16^ as subtropical and the region north of the Watase Line as temperate (Fig. 1). The region was split along this line because the biogeography of this region is often split between Yakushima and the Amami Islands, and the Kuroshio Current passes through this region.

## Results

### The *Acropora hyacinthus* lineage used in this study

**Figure 2:**
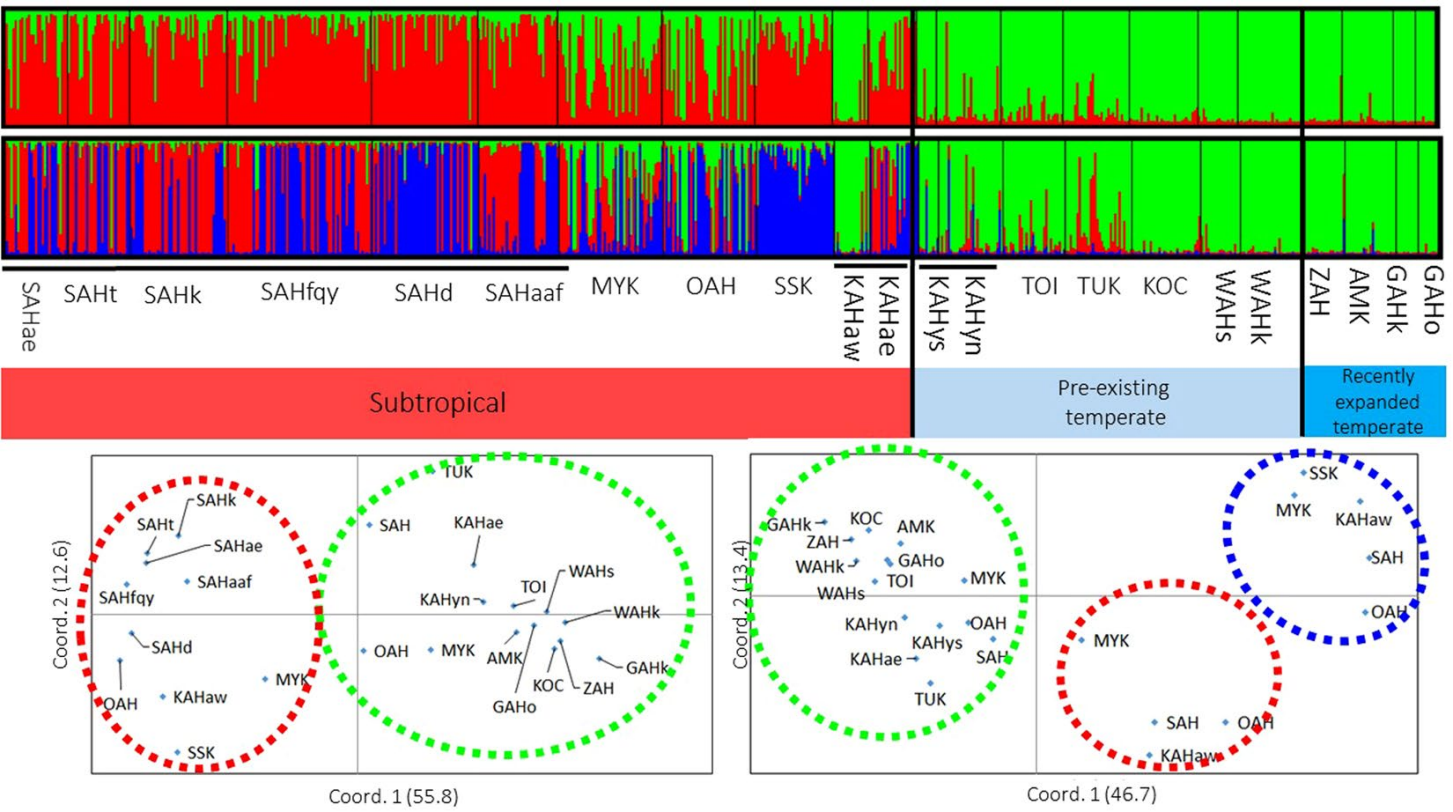
Bar plot data from STRUCTURE analysis (above *K* = 2, below *K* = 3) across all collected samples (above). The X-axis indicates individuals from different populations, and the Y-axis indicates the estimated proportions of different clusters shown in different colours. The PCoA plots are based on the populations separated into cryptic lineages revealed by STRUCTURE analysis at *K* = 2 and 3 (below). (**Left**) The first two coordinates explain 55.8% (x-axis) and 12.6% ((y-axis) of the data (*K* = 2); (**Right**) the first two coordinates explain 46.7% (x-axis) and 13.4% ((y-axis) of the data (*K* = 3).

Only 10366m5 has possible null alleles, and we thus excluded this locus from the genetic analysis of the population. A previous study indicated that there are cryptic species (lineages) in *Acropora hyacinthus* that are morphologically indistinguishable^10^. Thus, we identified the cryptic lineages in our dataset using STRUCTURE analysis^17^ to determine the lineage associated with the recently expanded populations in temperate regions. After removing 44 possible clones, our STRUCTURE analyses based on 645 multilocus genotypes (MLGs) indicated that the most likely number of clusters was *K* = 2, followed by 3 (Suppl. 1). At *K* = 2, temperate populations and some of the subtropical individuals were assigned to the same cluster (green lineage in Fig. 2), and the other lineage was restricted to south of Miyazaki. At *K* = 3, the subtropical specific cluster at *K* = 2 (red in Fig. 2) was further split into two different clusters (red and blue in Fig. 2). The principal coordinate analysis (PCoA) results also supported different lineages (Fig. 2). Moreover, populations with two or three different genetic lineages belonged to different clades in the neighbour-joining (NJ) phylogenetic trees (Suppl. 2). These results, as well as those of a previous study^11^, indicated that these populations are possibly different species^10^. Because we are interested in the genetic connectivity and diversity of the lineages that have recently expanded in temperate regions, we focused on one lineage (green lineage in Fig. 2) found in both temperate and subtropical regions. Individuals assigned to the green lineage with probabilities ≥70% in both the *K* = 2 and *K* = 3 results were used for the following analysis (Table 1). A relatively low value ≥70% was chosen because that lineage was rather rare in the subtropical region and many individuals found in the subtropical area are partially admixed with other lineages. Populations with ≥9 MLGs were used for subsequent analysis because smaller sample sizes are not suitable for population genetic analysis^18^. The final dataset included 240 individuals from 13 populations (shown in bold in Table 1). Initially, significant linkage disequilibrium was found in two pairs of loci (11401m4 and Amil2-23; 11401m4 and Amil2-2). However, linkages disappeared in the final dataset, which included only one lineage and no clones.

**Table 1:** Details of the samples used in this study.

	Region	Sampling site	Code	N	Depth (m)	Coordinates		MLG	1-MLG/N (%) Percentage of shared MLGs among samples	N. of Green lineage	N. of blue/red lineage (Fig. 2)
recently colonized in temperate area	Goto Islands	Oushima	GAHo	19	0.3~6.0	32°33′ 55.47″ N	128°53′ 56.57″ E	9	0.526	**9**	0
		Kuroshima	GAHk	13	4.1~5.6	32°35′ 56.18″ N	128°50′ 04.05″ E	10	0.231	**10**	0
	Kumamoto	Amakusa	AMK	25	N.A.	32°33′ 16.16″ N	129°59′ 59.91″ E	23	0.080	**23**	0
	Izu Islands	Shikinejima	ZAH	18	0.8~6.6	34°21′ 36.44″ N	139°12′ 02.39″ E	18	0.000	**17**	1
pre-existing temperate area	Wakayama	Kushimoto	WAHk	34	2.4~5.5	33°28′ 27.36″ N	135°44′ 26.92″ E	29	0.147	**29**	0
		Shirahama	WAHs	18	0.7~9.4	33°40′ 59.81″ N	135°20′ 11.41″ E	18	0.000	**18**	0
	Kochi	Tatsukushi	KOC	32	1.0~6.0	32°45′ 49.33″ N	132°51′ 38.39″ E	31	0.031	**30**	1
	Miyazaki	Tsukijima	TUK	46	N.A.	31°28′ 39.18″ N	131°23′ 14.67″ E	30	0.348	**23**	7
		Toimisaki	TOI	29	N.A.	31°21′ 00.47″ N	131°16′ 59.92″ E	28	0.034	**25**	3
	Yakushima	Tsukasaki	KAHyn	30	4.4~6.3	30°25′ 55.50″ N	130°34′ 34.46″ E	29	0.033	**24**	5
		Nanase	KAHys	13	5.6~7.7	30°14′ 42.24″ N	130°25′ 31.24″ E	9	0.357	8	1
subtropical area	Amami Islands	Saneku	KAHaw	22	1.5~4.8	28°11′ 33.24″ N	129°11′ 30.78″ E	22	0.000	0	22
		Sakibaru	KAHae	16	N.A.	28°19′ 54.83″ N	129°33′ 19.76″ E	16	0.000	**14**	2
	Okinawa Island	Sesokojima	SSK	35	N.A.	26°37′ 51.43″ N	127°51′ 23.57″ E	35	0.000	0	35
		Oura	OAH	42	N.A.	26°30′ 21.70″ N	128°02′ 28.84″ E	42	0.000	**9**	33
	Miyakojima	Yoshino	MYK	47	N.A.	24°47′ 57.72″ N	125°20′ 59.22″ E	47	0.000	**9**	38
	Sekisei Lagoon	Uro	SAHaaf	36	1.8~4.2	24°15′ 28.63″ N	124°10′ 39.49″ E	36	0.000	0	36
		Hanari East	SAHd	49	1.0~6.6	24°13′ 20.22″ N	123°56′ 16.41″ E	48	0.020	1	47
		Yonara Chanel	SAHfqy	65	1.0~3.8	24°22′ 00.32″ N	123°57′ 03.14″ E	65	0.000	0	65
		Kohamajima East	SAHk	44	0.5~2.8	24°21′ 06.98″ N	124°00′ 41.96″ E	44	0.000	0	44
		Haimi South	SAHt	28	2.2~4.8	24°16′ 06.23″ N	123°50′ 06.66″ E	28	0.000	1	27
		Aragusuku East	SAHae	28	2.8~4.7	24°15′ 07.03″ N	123°58′ 25.77″ E	28	0.000	2	26

The numbers of samples used in the population genetic analysis are shown in bold. N, total number of samples collected from each site. MLG, number of multilocus genotypes. N. of green lineage, number of green lineage samples; N. of blue/red lineage, number of blue/red lineage samples (Fig. 2).

N.A. not available because of the lack of depth data

### Genetic diversity

Possible clones with a probability of identity of *P* < 0.001 were more frequently found in the temperate region than in the subtropical region (9 out of the 11 populations in the temperate region but none in the subtropical region), which indicated that asexual reproduction (such as fragmentation) likely occurred more frequently in the temperate region than in the subtropical region (Table 1). High clonal rates (23.1% for GAHk and 52.6% for GAHo) were found in two Goto Island populations, both of which are recently expanded populations.

**Figure 3:**
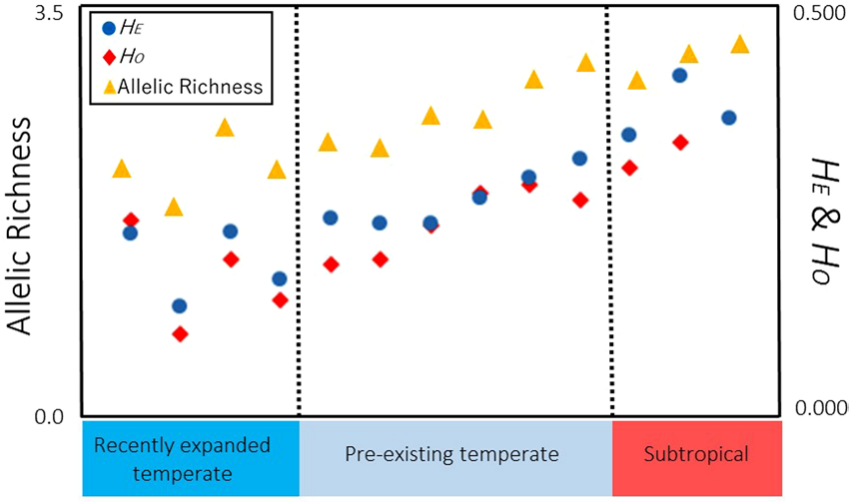
Plots of genetic diversity along central-to-peripheral populations. Circles indicate *H*_*E*_, expected heterozygosity, diamonds indicate *H*_*O*_, observed heterozygosity, and triangles indicate allelic richness.

After removing possible clones, the north peripheral populations had reduced genetic diversity, which was observed in particular in the recently expanded populations (Fig. 3, Suppl. 3). Genetic diversity metrics (allelic richness and heterozygosity) were more significantly reduced in the recently expanded populations than in the rest of the pre-existing temperate populations (Wilcoxon test, *P* = 0.010 and *P* = 0.038 for allelic richness and expected heterozygosity, respectively) (Fig. 3). Similarly, the genetic diversity metrics were significantly higher in subtropical populations than temperate ones (Wilcoxon test, *P* = 0.007 and *P* = 0.028 for allelic richness and expected heterozygosity, respectively). Notably, all temperate populations, except for one north Yakushima population (KAHyn), had more than one fixed (monomorphic) locus. All the recently expanded populations had 2 to 4 fixed loci out of 8 loci (Suppl. 3). In total, 24 individuals had private alleles. Eight out of the 32 individuals (25.0%) examined in the subtropical populations had private alleles, and 11 out of the 149 (7.4%) individuals in the pre-existing temperate populations had private alleles. Two out of 59 individuals (3.4%) were found to have private alleles in the recently expanded populations (One from Goto Island, GAHo, and another from Amakusa, AMK). These results suggest that subtropical populations have more private alleles than temperate populations, and recently expanded populations possibly originated from a small number of individuals with low genetic diversity.

### Isolation by distance (IBD) pattern and genetic connectivity

**Figure 4:**
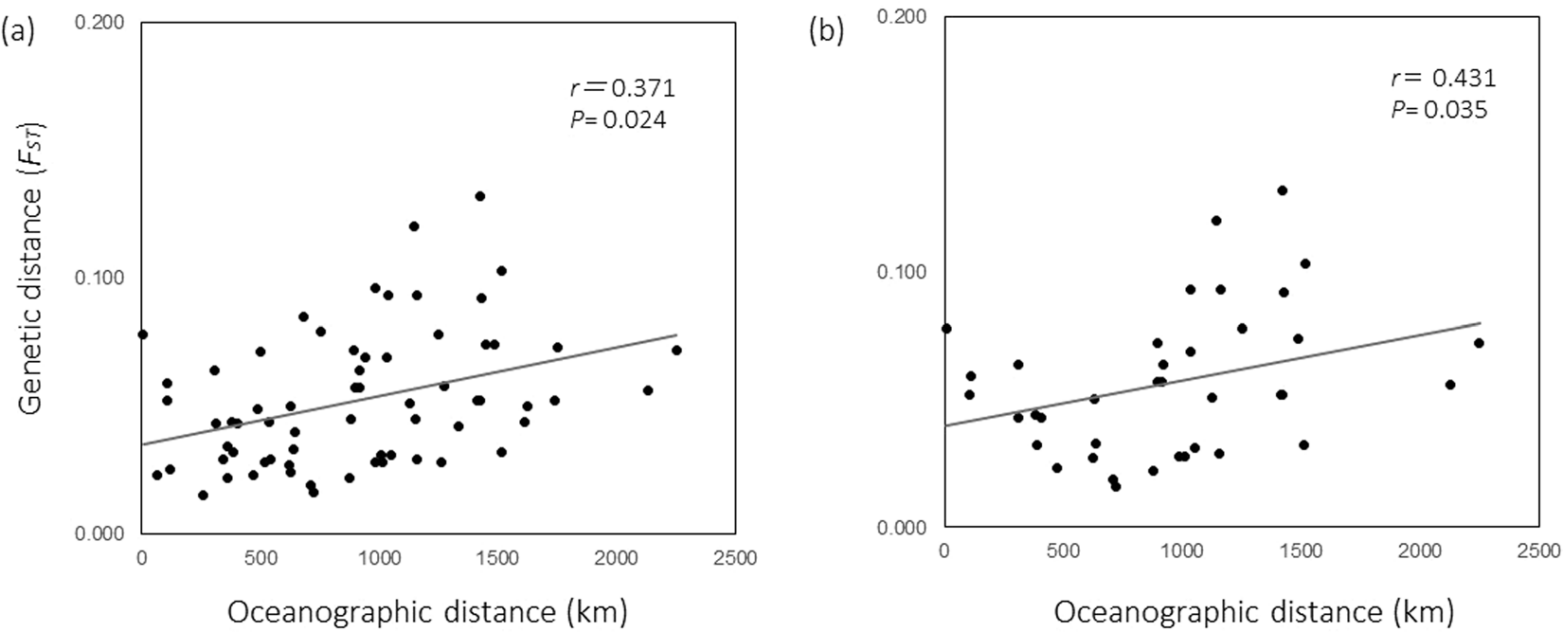
IBD patterns based on oceanographic distances using different sample sets ((**a**) all populations; (**b**) excluding recently colonized populations).

Significant IBD patterns within the green lineage were detected only when regressed onto oceanographic distance (*P* < 0.05) (Fig. 4, Suppl. 4). A weak IBD tendency was detected using straight-line distance (*P* = 0.085) only when the recently expanded populations were excluded, which possibly occurred because the recently expanded populations are not in genetic equilibrium.

The analysis of molecular variance (AMOVA) results suggested overall significant genetic structuring within the *A*. *hyacinthus* lineage that was examined in this study (global *F*_*ST*_ = 0.108, *P* < 0.001, Table 2). Significant genetic differentiation between the temperate and subtropical populations was not found, either including (all temperate vs subtropical, *F*_*CT*_ = 0.042, *P* = 0.064) or excluding (pre-existing temperate vs subtropical, *F*_*CT*_ = 0.024, *P* = 0.163) the recently expanded populations. The AMOVA results revealed non-significant genetic differentiation between the recently expanded populations and the pre-existing temperate populations (*F*_*CT*_ = −0.002, *P* = 0.424) and between the populations along the main stream of the Kuroshio Current vs the Tsushima Current (*F*_*CT*_ = 0.004, *P* = 0.275) in the temperate region (Table 2). This result suggests that populations in temperate regions were generally genetically close to each other. Pairwise tests showed almost no genetic differentiation among the recently expanded populations, whereas there were some significantly differentiated pairs among the pre-existing populations (Table 3). The Tsukijima population in Miyazaki (TUK) was one of the most isolated populations in the temperate region, most likely because the artificial structure surrounding this population (N. Yasuda pers. obs.) hindered larval exchange.

**Table 2:** Results of AMOVA with different scenarios using only the green lineage in Fig. 2. *P* < 0.05 are shown in bold.

Scenario	Source of variation	df	%var	*F*		*P*
Global test	Among populations	12	10.8	*F*_*ST*_	0.108	**<0**.**001**
	Within populations	467	89.2			
Temperate vs subtropical	Among groups	1	4.2	*F*_*CT*_	0.042	0.064
	Among populations within groups	11	9.4	*F*_*SC*_	0.098	**<0**.**001**
	Within populations	467	86.4	*F*_*ST*_	0.136	**<0**.**001**
Pre-existing temperate vs subtropical	Among groups	1	2.4	*F*_*CT*_	0.024	0.163
	Among populations within groups	7	9.9	*F*_*SC*_	0.102	**<0**.**001**
	Within populations	353	87.7	*F*_*ST*_	0.123	**<0**.**001**
Recently vs pre-existing in temperate	Among groups	1	0.07	*F*_*CT*_	0.001	0.416
	Among populations within groups	8	10.5	*F*_*SC*_	0.105	**<0**.**001**
	Within populations	406	89.46	*F*_*ST*_	0.105	**<0**.**001**
Kuroshio vs Tsushima Current in temperate	Among groups	1	0.07	*F*_*CT*_	0.001	0.420
	Among populations within groups	8	10.47	*F*_*SC*_	0.105	**<0**.**001**
	Within populations	406	89.5	*F*_*ST*_	0.105	**<0**.**001**

**Table 3:** Pairwise *F*_*ST*_, *P* values for *F*_*ST*_, *D*_*EST*_ and *P* values for Fisher’s exact test. Significant tests after sequential Bonferroni correction are shown in bold.

		Recently colonized in temperate area				Pre-existing in temperate area				Subtropical area				
		GAHo	GAHk	AMK	ZAH	WAHk	WAHs	KOC	TUK	TOI	KAHyn	KAHae	OAH	MYK
***F***_***ST***_ **(above)** ***P*** **value (below)**														
Recently colonized in temperate	GAHo		0.073	0.056	0.050	0.060	0.054	0.048	**0**.**102**	0.031	0.041	**0**.**084**	0.085	0.065
	GAHk	0.010		0.050	0.050	0.054	**0**.**067**	0.026	**0**.**169**	0.042	**0**.**060**	**0**.**117**	**0**.**107**	0.086
	AMK	0.016	0.001		0.031	0.027	0.028	0.016	**0**.**128**	0.023	**0**.**041**	**0**.**094**	**0**.**072**	0.049
	ZAH	0.009	0.003	0.012		0.032	0.019	0.022	**0**.**108**	0.030	0.028	**0**.**069**	0.067	0.053
Pre-existing in temperate	WAHk	0.013	0.001	0.041	0.003		0.023	**0**.**022**	**0**.**118**	0.028	**0**.**038**	**0**.**087**	0.068	0.047
	WAHs	0.012	<0.001	0.047	0.257	0.097		**0**.**028**	**0**.**099**	0.027	0.023	**0**.**065**	0.049	0.042
	KOC	0.002	0.003	0.039	0.002	<0.001	<0.001		**0**.**105**	0.014	**0**.**032**	**0**.**079**	**0**.**068**	0.041
	TUK	**<0**.**001**	<0.001	<0.001	<0.001	<0.001	<0.001	<0.001		0.068	**0**.**062**	**0**.**087**	**0**.**087**	**0**.**100**
	TOI	0.313	0.008	0.034	0.003	0.003	0.003	0.092	0.000		0.024	**0**.**066**	0.055	0.043
	KAHyn	0.006	0.001	<0.001	0.030	<0.001	0.052	<0.001	<0.001	0.002		0.027	0.030	0.043
Subtropical area	KAHae	**<0**.**001**	<0.001	<0.001	<0.001	<0.001	<0.001	<0.001	<0.001	<0.001	0.008		0.042	**0**.**074**
	OAH	0.003	0.001	0.001	0.006	0.002	0.079	<0.001	<0.001	0.002	0.162	0.025		0.046
	MYK	0.024	0.003	0.041	0.019	0.059	0.065	0.014	<0.001	0.028	0.015	<0.001	0.167	
***DEST*** **Fisher’s exact test**														
Recently colonized in temperate	GAHo		0.022	0.024	0.040	0.022	0.039	0.030	0.046	0.009	0.038	0.079	0.054	0.034
	GAHk	0.061		0.025	0.021	0.023	0.025	0.004	0.045	0.009	0.014	0.062	0.039	0.042
	AMK	**<0**.**001**	0.011		0.013	0.004	0.009	0.012	0.012	0.010	0.030	0.064	0.050	0.013
	ZAH	**<0**.**001**	0.024	0.032		0.011	0.003	0.012	0.032	0.022	0.005	0.029	0.026	0.016
Pre-existing in temperate	WAHk	**<0**.**001**	0.050	0.018	0.082		0.000	0.016	0.016	0.016	0.026	0.056	0.032	0.004
	WAHs	**<0**.**001**	0.002	0.003	0.485	0.127		0.011	0.021	0.012	0.007	0.021	0.017	0.000
	KOC	**<0**.**001**	0.271	**0**.**001**	0.030	**<0**.**001**	**<0**.**001**		0.024	0.000	0.009	0.044	0.031	0.019
	TUK	**<0**.**001**	**<0**.**001**	**<0**.**001**	**<0**.**001**	**<0**.**001**	**<0**.**001**	**<0**.**001**		0.011	0.035	0.044	0.041	0.025
	TOI	0.025	0.141	0.026	0.004	**<0**.**001**	**<0**.**001**	0.209	**<0**.**001**		0.013	0.039	0.024	0.022
	KAHyn	**<0**.**001**	0.009	**<0**.**001**	0.016	**<0**.**001**	0.002	**<0**.**001**	**<0**.**001**	**<0**.**001**		0.009	0.009	0.018
Subtropical area	KAHae	**<0**.**001**	**<0**.**001**	**<0**.**001**	**<0**.**001**	**<0**.**001**	**<0**.**001**	**<0**.**001**	**<0**.**001**	**<0**.**001**	0.024		0.022	0.014
	OAH	**0**.**001**	0.003	**<0**.**001**	**<0**.**001**	**<0**.**001**	0.006	**<0**.**001**	**<0**.**001**	**0**.**001**	0.126	0.056		0.011
	MYK	**<0**.**001**	**<0**.**001**	**<0**.**001**	**<0**.**001**	**<0**.**001**	**0**.**001**	0.005	**<0**.**001**	0.003	**<0**.**001**	**<0**.**001**	0.154	

**Figure 5:**
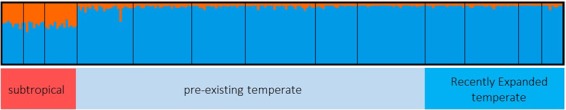
Bar plot at *K* = 2 using the LOCPRIOR model (temperate vs subtropical, green lineage only).

STRUCTURE analysis was again performed within the green lineage with and without prior information (subtropical vs temperate). The results indicated that *K* = 1 best explained the data (Suppl. 5a,b), which suggests admixture and no clear genetic divergence between the temperate and subtropical regions, and these findings are consistent with the AMOVA results. However, when using the LOCPRIOR model, different proportions of assigned lineages were found between temperate and subtropical regions at *K* = 2 (Fig. 5) with a small *r* value (0.869), which implies that very small genetic differences exist between the two regions.

The estimation of recent gene flow based on the assignment method using GeneClass2 showed that 95 out of 240 individuals were successfully assigned to one of the populations. Most of the successfully assigned individuals (15 out of 16) in the recently expanded populations suggested self-seeding. Only one out of 76 (1.3%) temperate individuals were possible recent immigrants from subtropical populations (Suppl. 6).

### Particle tracking simulation

**Figure 6:**
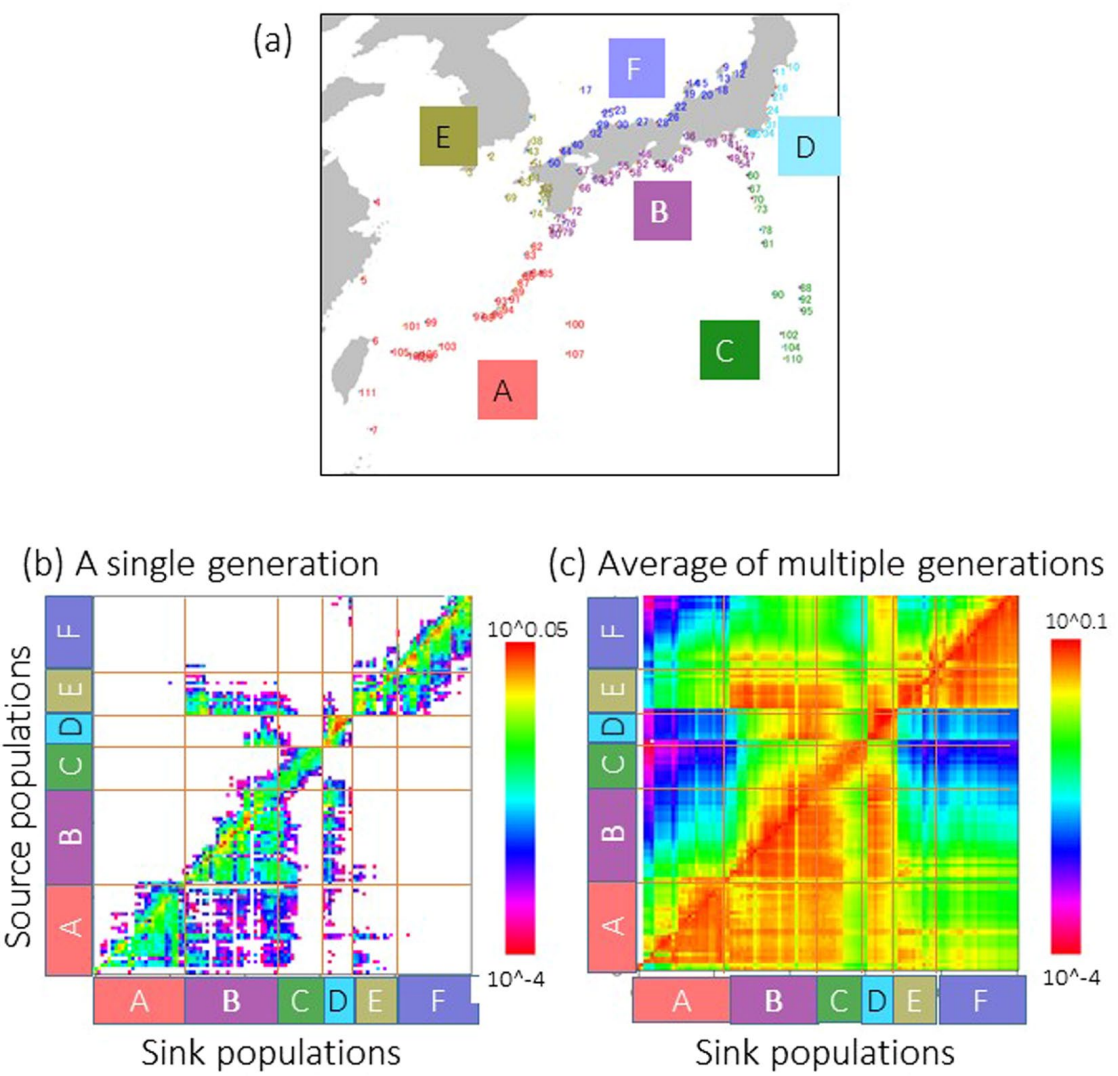
(**a**) Sites of simulated larvae release. The region codes A~F correspond to A~F in (**b, c**) and (**b**) connectivity matrix based on the calculation of 23 years of larval dispersal simulations. (**c**) Connectivity matrix after iterating dispersal over 1000 generations. The colour contours on the right indicate the probability of a larva arriving at a sink site from a source site.

Larval dispersal generally occurred northward along the Kuroshio Current (Fig. 6b,c). Larval dispersal from temperate to subtropical areas seemed uncommon within a single generation because larval dispersal is physically limited to the path of the Kuroshio Current (here called the Kuroshio-associated barrier), although dispersal could be achieved through multiple generations or occasional long dispersals (Fig. 6c). The Kuroshio-associated barrier corresponds to the biogeographic boundary called the Watase Line (Fig. 1). Unidirectional dispersal was observed from the western Kyushu region (E in Fig. 6a) along the Tsushima Current to the temperate region along the southern Japanese coast (B in Fig. 6a) along the main stream of the Kuroshio Current (Fig. 6c).

## Discussion

Cryptic lineages were found in *A*. *hyacinthus*, while only one lineage is dominant in the temperate region and is apparently expanding to the north. The Kuroshio-associated barrier found in the numerical simulation and/or the lack of adaptability to the temperate environment could hinder northward migration of the blue/red lineages (Fig. 2). The Kuroshio-associated barrier also matched the Watase Line, which is the biogeographic barrier that separates terrestrial fauna and was caused by land bridges and sea barriers in past climates^16^. Larval dispersal simulations using particle tracking averaged over 24 years indicated discontinuous patterns of connectivity between Yakushima and the Amami Islands (Fig. 6). This discontinuity is possibly due to the Kuroshio path crossing between Yakushima and the Amami Islands, which prevents direct dispersal of single generations from the subtropics to areas further north (referred to as the Kuroshio-associated dispersal barrier). This study demonstrates that the Watase Line can be found in marine species due to the existence of the Kuroshio-associated dispersal barrier. The limited larval transport from subtropical to temperate regions within a single generation (Fig. 6) suggested that the probability of future poleward migration of the other lineages (red/blue in Fig. 2) of *A*. *hyacinthus* would depend on whether the larvae of these lineages could pass through this barrier via long or multi-generation dispersal. Further regular monitoring of the distributions of all cryptic lineages using genetic markers is necessary to assess the poleward migration of the species.

Reduced genetic diversity (heterozygosity, allelic richness, and clonal rate) was observed in the recently expanded *Acropora hyacinthus* populations distributed near the edge of the species’ range. One historical interpretation is that reduced genetic diversity near the edge of a species’ range is the result of greater isolation in peripheral populations than in core populations where species are more abundant than along the periphery^8^. However, this study did not detect a gradual increase in genetic isolation towards peripheral populations (Table 3). Alternatively, IBD patterns produced by a simple linear stepping-stone model explains the reduced genetic diversity near the edge. If a population structure is assumed to exhibit linearly uniform distribution over a large distance with limited migration in a simple linear stepping-stone model, then IBD genetic structure can theoretically be observed^19^. Under such a situation, Tajima^19^ demonstrated that the level of DNA polymorphism is highest in the central populations and gradually decreases towards peripheral populations. Our data relatively fit this linear stepping-stone model because significant IBD patterns were observed when oceanographic distance was used. Although more data are needed, a linear stepping-stone model can at least partially explain the observed patterns. Lastly, it is also possible that genetic diversity will decrease towards the edge of a species’ range in response to different selection pressures^20^, although there is an opposing view that a stressful peripheral environment can increase the number of genotypes in some cases^21^. Environmental factors, such as low sea water temperature^22^, high concentration of chlorophyll a, particulate organic carbon (POC)^23^, reduced aragonite saturation^24^, light intensity^25^, and high turbidity^26^ around northern populations (Suppl. 7), could affect the suitability of reef-building corals habitats^25^ and subsequently erode genetic diversity. On the other hand, the larger genetic diversity in the subtropical populations can be partly explained by possible hybridization between closely related lineages (as found in Fig. 2).

Some previous studies have also provided evidence for reduced genetic diversity in peripheral coral populations. For example, both brooder and broadcaster coral species in the southernmost reef in the Pacific showed lowered genetic diversity^27^. In the Atlantic, another coral species, *Montastraea cavernosa*, showed lower genetic diversity in peripheral populations^28^. These studies suggested that a small founding population, small effective population size of genetic drift, or inbreeding depression caused reduced genetic diversity in peripheral coral populations. However, expanding coral populations do not always follow this trend of reduced genetic diversity, as was demonstrated in the Mediterranean coral *Oculina patagonica*^29^.

The clonal rates were higher in temperate regions than in subtropical regions. Although the mechanism is unclear, different habitat characteristics (i.e., reef structure in subtropical regions and non-reef structure in temperate regions) potentially caused different rates of sexual reproduction success in *A*. *hyacinthus*. In *Acropora palmata*, different rates of asexual reproduction are known to be related to different habitat characteristics^30^.

While the historical gene flow analysis (e.g., *F*-statistics; Tables 2 and 3, Suppl. 5) showed overall genetic similarities among temperate populations, contemporary gene flow analysis (assignment tests) indicated that all studied populations, including those in the peripheral temperate regions, are basically self-seeding (Suppl. 6). This result concords with the fact that peripheral temperate coral populations are mostly self-sustained by local spawning^31^. Recently expanded populations were therefore possibly seeded from other temperate populations by occasional long dispersal and then sustained themselves by self-seeding.

Although the recently expanded Goto populations (GAHo) had low genetic diversity and a small number of samples was analysed (MGL = 9), one individual had a private allele. One possible explanation is that harsh environments around GAH (Suppl. 7) caused some strong directional selective pressures on the coral populations, which reduced the genetic diversity and changed the allele frequencies, as was discussed above (selective pressure increased the frequency of rare alleles, and a private allele was found). Alternatively, the Goto population was colonized by other genetically diverged populations, such as those from Taiwan through the Taiwan-Tsushima warm current system^32^, that were not analysed in this study. Previous study has claimed that peripheral populations with low genetic diversity are some of the most active regions of speciation because of such genetically distinct features^33^. Further genetic analysis of Goto will be interesting to examine potential speciation in such a peripheral region.

Significant IBD patterns were detected only when oceanographic distance was used (Fig. 4), which indicates that pairwise genetic distances (*F*_*ST*_) significantly increased with geographic distance. This result indicates that overall, the effect of genetic drift is stronger than the effect of gene flow between distant populations.

We expected that the larval dispersal model rather than oceanographic distance would have significant IBD patterns because genetic structure is considered to be most associated with larval dispersal. However, the IBD patterns that were derived using the number of larvae between sites based on numerical simulations did not improve in comparison to those using Euclidean distances and oceanographic distances (Fig. 4, Suppl. 4). This result might be because the genetic markers used in this study captured historical events rather than contemporary gene flow, especially in the *F*_*ST*_ calculations, which assume equilibrium. Similarly, the genetic distance of sea stars across the Indo-Pacific region was best explained by Euclidean distance and not modelled larval distance in a previous study^34^ in which the authors considered that the estimated *F*_*ST*_ reflected historical gene flow rather than contemporary gene flow. Another explanation for the weak correlation with IBD patterns is the lack of biological characteristics (i.e., larval behaviour) in the larval dispersal models used in this study. A more sophisticated model that includes the biological aspects of *A*. *hyacinthus* would improve the correlation with genetic distance, as in a previous study^35^.

Although corals in subtropical regions are threatened due to climate change, they are expanding and propagating into temperate regions^3^. Many climate change predictions of the species distributions of corals show a general loss of reef habitats in many areas except for the relatively robust eastern marginal Pacific region, including temperate Japan^36^. Given the moderate levels of neutral genetic diversity and the persistence and increase in coral cover before and after the rise in sea water temperatures in pre-existing temperate coral populations, we propose that some of the pre-existing temperate regions in Japan would be good refugia for at least one of the *A*. *hyacinthus* lineages. Pre-existing temperate regions are indeed the potential habitats that “part of biodiversity retreat to, persist in and can potentially expand” under climate change conditions. In temperate regions, seaweed beds have been replaced by temperate corals, including *A*. *hyacinthus*, and the coral cover is currently expanding in these regions^37^. Although a direct association between neutral genetic diversity and resilience of coral populations against environmental change is partly under debate^8^, some coral genotypes are known to be associated with stress tolerances such as disease and higher water temperature^38^. Thus, moderate genetic diversity in pre-existing temperate populations may indicate stability and higher tolerance against unexpected extreme environmental changes such as local anthropogenic stress. Nonetheless, much less attention has been paid to coral assemblages and their conservation in temperate regions than in subtropical regions. Unlike in subtropical regions, where tourism, recreation, and fisheries are directly associated with the abundance of corals, temperate corals are sometimes regarded as a nuisance by local fishermen in temperate regions because subtropical fishes are of low economic value in these areas. In addition, expensive fishing nets become entangled on and broken by hard corals, causing pecuniary damage to fishermen (N. Yasuda personal communication with local fishermen in Miyazaki). Because reef-building corals help to increase overall biodiversity^39^ by providing food and habitats with complex structures in coastal ecosystems, there is a need to review the value of temperate corals for conservation.

The importance of conserving the pre-existing temperate corals discussed in this study does not dismiss or discourage the conservation of subtropical corals. Rather, two out of the three *A*. *hyacinthus* lineages were found in only the subtropical region, and the highest genetic diversity was found in the subtropical region, indicating that these areas are nonetheless important.

This study indicated that recently expanded marginal populations could have low genetic diversity and are possibly more vulnerable to environmental changes than pre-existing populations. This finding underpins the importance of assessing the genetic diversity of populations that have recently expanded due to climate change for conservation purposes, in particular when anticipating the roles of these areas as refugia under climate change.

## Methods

### Coral sampling and genotyping

In total, 689*A*. *hyacinthus* samples were collected using SCUBA from 22 populations along the Kuroshio and Tsushima Currents (Table 1, Fig. 1). This study included four temperate populations that have recently expanded possibly due to climate change: two populations from Goto Island and the Amakusa and Shikine populations^3^. No records of *A*. *hyacinthus* were found in Goto and Amakusa in a 1965–1977 survey. There was no existing record of *A*. *hyacinthus* in Shikine in a 1986 survey^3^, but we discovered large colonies in 2016 in this study. Each sample was photographed except for the AMK, KOC, MYK, SSK, TUK, TOI, and OAH specimens (Table 1). Wherever possible about 5 cm of skeleton specimens were collected and deposited at the University of Miyazaki (MUFS-Acr101–310, 371–428, 500–531, 569–717, 768–801). Species identification was further confirmed by Dr. Hironobu Fukami based on morphological information.

Genomic DNA was extracted using a modification of a previously published method^40^. Genotypes of the corals were determined by 8 microsatellite loci^41,42^. Eight loci were amplified in 2 multiplex PCR sets using different dyes and non-overlapping loci (Plex1 includes 8346m3, 11401m4, 8499m4, and 10366m5; Plex2 included Amil2-2h, Amil2-23h, Amil2-10, and Amil2-12). For Amil2-2h and Amil2-23h, we used modified primers to increase PCR efficiency for *A*. *hyacinthus* (Suppl. 8). We used an 8 μl reaction containing 1 μl of template DNA, 0.03 μl of each primer (50 μM), 4 μl of Type It (Microsatellite Kit, Qiagen), and 2.94 μl of water. The thermal cycler profile that was used had an initial denaturation step at 95 °C for 3 min followed by 30 cycles of 95 °C/30 s, 50 °C/30 s, 72 °C/30 s, with a final extension step at 72 °C for 5 min.

The multiplexed PCR product was diluted by 100, and 1 μl of the diluted PCR product was added to 10 μl of highly deionized formamide containing 0.15 μl of the GeneScan-600 (LIZ) size standard (Applied Biosystems, Foster City, CA, USA) for genotyping on an ABI 3730xl automated sequencer (GeneMapper® ver. 5, Applied Biosystems), which was used to determine the fragment sizes (alleles) of all samples. All genotyping was manually checked. Whenever we found ambiguous patterns or no peak, we re-extracted the genomic DNA and re-amplified it or removed the sample from the analysis. We tested for the presence of null alleles using MICRO-CHECKER ver. 2.2.3^43^, and the locus positive for null alleles was excluded from the genetic analysis of the population.

The clonal structure of each coral population was examined by identifying the same MLGs based on 8 microsatellite loci; these were identified as possible clones (asexually reproduced by fragmentation), and all except one were excluded from subsequent analyses. The inbreeding coefficient of a population (*F*_*IS*_), was evaluated with GenAlEx ver. 6.502^44^. Genotypic linkage disequilibrium across 8 loci was examined using GENEPOP on the web (ver. 4.0.10)^45^ by estimating exact *P* values using the Markov chain method with 10,000 dememorization steps, 1,000 batches, and 10,000 iterations per batch.

Different measures of genetic diversity were estimated for each population, including the number of alleles per locus (Na), the observed (H_O_) and expected (H_E_) heterozygosity using GenAlEx ver. 6.502^44^, and allelic richness (A) using FSTAT ver. 2.932^46^.

### Identifying cryptic lineages distributed in both temperate and subtropical regions

We first identified the cryptic lineages of *A*. *hyacinthus* found in a previous study^11^. STRUCTURE analysis was conducted using the admixture model without prior information. The Markov chain Mote Carlo (MCMC) chains used a burn-in of 500,000 chains followed by 500,000 MCMC replications. Ten independent chains were run for each *K* = 1 to 10 to confirm convergence. The coefficient of ancestry was calculated and visualized for each individual across 10 runs for the most likely values of *K* (Delta *K*^47^), in CLUMPAK^48^ Among the lineages identified in the STRUCTURE analysis above, we used only the lineage distributed in both temperate and subtropical regions in subsequent analyses (green lineage in Fig. 2). PCoA was conducted to confirm the different lineages found in the STRUCTURE analysis using GenAlEx ver. 6.502^44^. A NJ phylogenetic tree was reconstructed using Nei’s genetic distance^49^ as implemented in POPULATIONS ver. 1.2.32^50^ with 1000 bootstrap values and visualized this tree using TreeViewX ver. 0.5.0^51^.

STRUCTURE was again run to examine the intra-green-lineage genetic structure. First, we analysed STRUCTURE without any prior information and then used prior information about different regions (temperate or subtropical) using the LOCPRIOR model^52^. All other STRUCTURE run settings were the same as described above.

### Genetic connectivity

To examine the degree of genetic connectivity between temperate and subtropical populations, population genetic structure was examined by AMOVA using Arlequin ver. 3.5^53^. After calculating global *F*_*ST*_, we evaluated the genetic structure using the following prior groupings: (1) all temperate populations vs subtropical populations, (2) recently expanded populations vs pre-existing temperate populations, and (3) populations along the temperate main stream of the Kuroshio Current (ZAH, WAHk, WAHs, KOC, TUK TOI, and KAH) vs populations along the Tsushima Current (AMK, GAHo, and GAHk). Pairwise *F*_*ST*_ and pairwise *D*_*EST*_ (Jost’s direct measure of differentiation^54^) values across populations were calculated to estimate relatively isolated populations using GenAlEx 6.503^44^. The significance of population differentiation was also determined based on Fisher’s exact test using Genepop on the web ver. 4.0.10^45^ with Markov chain parameters with 10,000 dememorization steps, 1,000 batches, and 10,000 iterations per batch. Sequential Bonferroni correction was applied to determine statistical significance for all pairs of populations (α < 0.05).

GeneClass2^55^ was used to estimate recent gene flow between temperate and subtropical populations using the criteria and the computational algorithm of Rannala and Mountain^56^ with default settings. Individuals were assigned to a source population when an individual had the highest assigned probability >70%, and others were regarded as unassigned. Because the two geographically close Wakayama populations (WAHs and WAHk) and the two Goto populations (GAHo and GAHk) were not significantly differentiated (Table 3), we combined these populations for only the GeneClass2 analysis.

#### IBD

To examine whether neighbouring populations have stronger gene flow than distant populations, the IBD patterns based on pairwise *F*_*ST*_ values were regressed onto (1) Euclidean distances between sites, (2) oceanographic distances avoiding physically impossible routes (e.g., passing on land) that were manually estimated using Google Earth and (3) simulated number of accumulated larvae based on a particle tracking model with iterations after 1000 generations (see below, Suppl. 10). We used two different datasets, one including all green lineage populations and the other excluding four recently expanded populations. Significance was tested using the Mantel permutation test (10,000 randomizations) using R ver. 3.4.2. Because the TUK population was surrounded by artificial structures that prevented larval dispersal, this population was excluded from the IBD analysis.

### Lagrangian particle tracking simulation

To estimate the probability of larval dispersal between different sites based on ocean current systems, we conducted Lagrangian particle tracking simulation using the Connectivity Modelling System (CMS)^57^ with Global HYCOM (Hybrid Coordinate Ocean Modelling^58^) ocean-current analysis/reanalysis products (https://hycom.org). CMS can evaluate a full range of transport and fate variabilities at high computational efficiency. In the particle tracking model, larvae are treated as passive particles transported on the surface by horizontal current velocities. The particles regarded as larvae were totally released from 111 habitat zones of 1/12° × 1/12° rectangles (~10 km^2^) (Fig. 6a). The timings of spawning (particle release) from each site were assumed to have begun on the first full moon before the integrated water temperature anomaly from 13 °C from February 1st exceeded 1000 °C day (∫max(T-13, 0)dt). The parameters for estimating the spawning dates (1 °C, February 1^st^, and 1000 °C·day) were determined by trial-and-error adjustment using previous records of *Acropora* spawning timing in Japan. The estimated spawning dates were in general agreement with the records of previous spawning timing (Aizawa *et al*. unpub. data). The time-series dataset of sea surface temperature used to estimate spawning timing at each site was obtained by extraction from the Global HYCOM analysis/reanalysis products. Spawning events were assumed to take place 3 days before the spawning full moon and to continue for 10 days. From each site, 50 particles were released each day, and a total of 500 particles were released in one year. The maximum larval dispersal period was set for 42 days, which is the maximum length of pelagic larval duration of *A*. *hyacinthus*. It was assumed that larvae could begin settling on coral habitats three days after particle release. When the particles passed by the coral habitats, the particles were counted as successful recruitments (the number of larvae dispersed from one place). Finally, a connectivity matrix was obtained by conducting the particle tracking simulation for a total of 24 years from 1993 to 2016. Using the average of the 24-year connectivity matrix, we iterated particle dispersal to simulate the long-term accumulation of dispersed larvae and consider the stepping-stone-like connectivity. This calculation was repeated until the number of the accumulated particle stabilized over time (1000 generations).

## Supplementary information

Suppl. 9

Suppl. material

## Data Availability

The datasets generated during and/or analysed during the current study are available in the Dryad repository (10.5061/dryad.5ps117g).
