# Supplementary material for: The potential role of temperate Japanese regions as refugia for the coral Acropora hyacinthus in the face of climate change
Source: Sci Rep. 2019 Feb 13;9:1892. doi: 10.1038/s41598-018-38333-5 (PMC6374466; doi:10.1038/s41598-018-38333-5)
Supplement: Supplementary file 2 — Suppl. material [file 41598_2018_38333_MOESM2_ESM.pdf]

Supplementary data

Title: The potential role of temperate Japanese regions as refugia for the coral *Acropora hyacinthus* in the face of climate change

Authors: Aki Nakabayashi, Takehisa Yamakita, Takashi Nakamura, Hiroaki Aizawa, Yuko F Kitano, Akira Iguchi, Hiroya Yamano, Satoshi Nagai, Agostini Sylvain, Kosuke M. Teshima, Nina Yasuda.

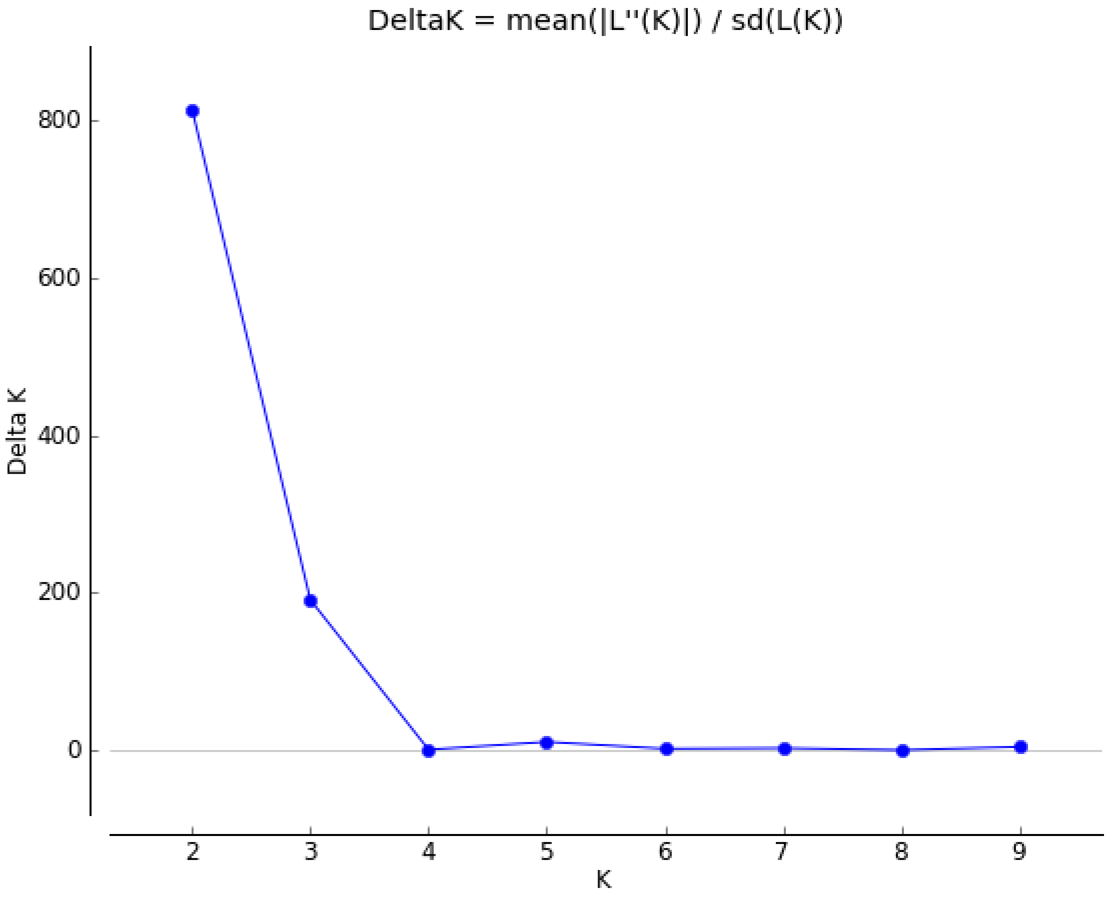

Suppl. 1 Delta *K* estimation of STRUCTURE analysis using whole data sets including cryptic lineages.

Suppl. 2 Neighbour-joining tree using Nei's genetic distance (Da) among populations after separating into cryptic lineages. (a) *K*=2 and (b) *K*=3. Numbers are bootstrap values.

(a)

(b)

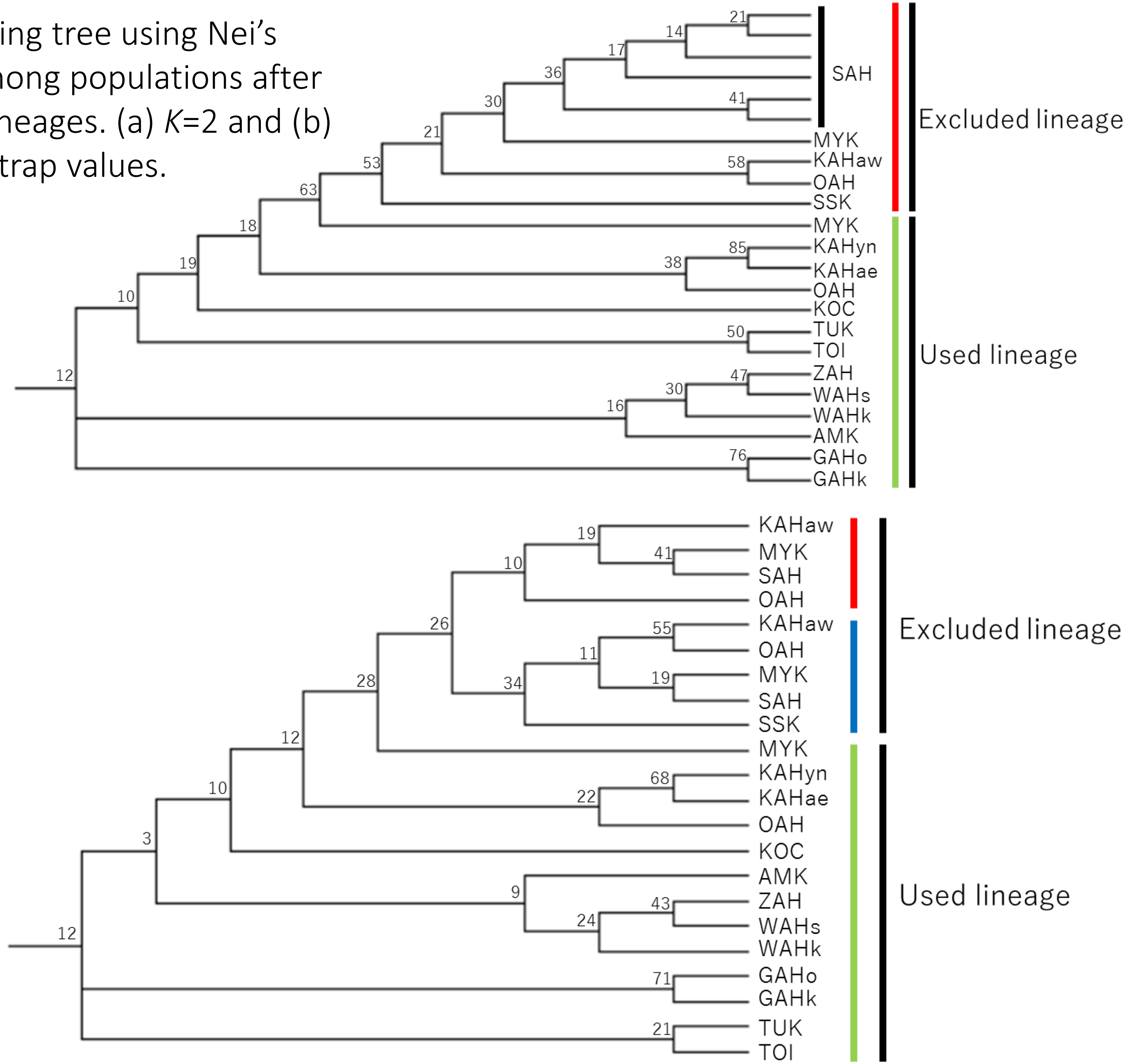

Suppl. 3 Genetic variability of each locus within each population. Number of samples (N); number of alleles (Na); number of effective alleles (Ne); Information Index (I); Observed (Ho); Expected (He) and Unbiased (UHe) Heterozygosities; and Fixation Index (F).

Pop	Locus	N	Na	Ne	I	Ho	He	UHe	F
GAHo	8346m3	9	2	1.246	0.349	0.222	0.198	0.209	−0.125
	11401m4	9	5	3.857	1.459	0.778	0.741	0.784	−0.050
	8499m4	9	1	1.000	0.000	0.000	0.000	0.000	#N/A
	Amil2-2	9	2	1.670	0.591	0.556	0.401	0.425	−0.385
	Amil2-23	9	1	1.000	0.000	0.000	0.000	0.000	#N/A
	Amil2-10	9	3	1.256	0.426	0.111	0.204	0.216	0.455
	Amil2-12	9	1	1.000	0.000	0.000	0.000	0.000	#N/A
	10366m5	9	4	2.348	1.014	0.111	0.574	0.608	0.806
GAHk	8346m3	10	1	1.000	0.000	0.000	0.000	0.000	#N/A
	11401m4	10	4	2.353	1.063	0.300	0.575	0.605	0.478
	8499m4	10	3	1.361	0.518	0.300	0.265	0.279	−0.132
	Amil2-2	10	2	1.105	0.199	0.100	0.095	0.100	−0.053
	Amil2-23	10	1	1.000	0.000	0.000	0.000	0.000	#N/A
	Amil2-10	10	1	1.000	0.000	0.000	0.000	0.000	#N/A
	Amil2-12	10	1	1.000	0.000	0.000	0.000	0.000	#N/A
	10366m5	10	6	3.704	1.495	0.300	0.730	0.768	0.589
AMK	8346m3	23	2	1.240	0.344	0.130	0.194	0.198	0.327
	11401m4	22	9	5.095	1.858	0.727	0.804	0.822	0.095
	8499m4	23	4	1.666	0.798	0.304	0.400	0.409	0.239
	Amil2-2	23	4	1.195	0.386	0.174	0.164	0.167	−0.064
	Amil2-23	23	1	1.000	0.000	0.000	0.000	0.000	#N/A
	Amil2-10	23	1	1.000	0.000	0.000	0.000	0.000	#N/A
	Amil2-12	21	1	1.000	0.000	0.000	0.000	0.000	#N/A
	10366m5	23	5	3.317	1.346	0.130	0.698	0.714	0.813
ZAH	8346m3	17	1	1.000	0.000	0.000	0.000	0.000	#N/A
	11401m4	15	6	2.332	1.242	0.333	0.571	0.591	0.416
	8499m4	16	2	1.205	0.311	0.188	0.170	0.175	−0.103
	Amil2-2	17	3	1.438	0.578	0.353	0.304	0.314	−0.159
	Amil2-23	17	1	1.000	0.000	0.000	0.000	0.000	#N/A
	Amil2-10	17	2	1.061	0.133	0.059	0.057	0.059	−0.030
	Amil2-12	17	2	1.061	0.133	0.059	0.057	0.059	−0.030
	10366m5	16	8	2.829	1.397	0.375	0.646	0.667	0.420
WAHk	8346m3	29	1	1.000	0.000	0.000	0.000	0.000	#N/A
	11401m4	29	7	3.602	1.502	0.517	0.722	0.735	0.284
	8499m4	28	6	2.469	1.210	0.429	0.595	0.606	0.280
	Amil2-2	29	3	1.482	0.616	0.310	0.325	0.331	0.046
	Amil2-23	29	1	1.000	0.000	0.000	0.000	0.000	#N/A
	Amil2-10	29	2	1.035	0.087	0.034	0.034	0.034	−0.018
	Amil2-12	29	1	1.000	0.000	0.000	0.000	0.000	#N/A
	10366m5	29	7	4.335	1.653	0.621	0.769	0.783	0.193
WAHs	8346m3	18	2	1.117	0.215	0.111	0.105	0.108	−0.059
	11401m4	18	6	2.711	1.283	0.389	0.631	0.649	0.384
	8499m4	18	4	1.600	0.746	0.222	0.375	0.386	0.407
	Amil2-2	18	3	1.742	0.730	0.500	0.426	0.438	−0.174
	Amil2-23	18	1	1.000	0.000	0.000	0.000	0.000	#N/A
	Amil2-10	18	2	1.117	0.215	0.111	0.105	0.108	−0.059
	Amil2-12	18	1	1.000	0.000	0.000	0.000	0.000	#N/A
	10366m5	16	6	4.971	1.681	0.500	0.799	0.825	0.374

Pop	Locus	N	Na	Ne	I	Ho	He	UHe	F
KOC	8346m3	30	3	1.070	0.169	0.067	0.065	0.066	−0.026
	11401m4	30	8	5.521	1.849	0.867	0.819	0.833	−0.058
	8499m4	29	6	1.679	0.859	0.483	0.404	0.411	−0.194
	Amil2-2	30	4	1.272	0.472	0.067	0.214	0.218	0.688
	Amil2-23	30	4	1.107	0.253	0.100	0.097	0.098	−0.034
	Amil2-10	30	2	1.034	0.085	0.033	0.033	0.033	−0.017
	Amil2-12	30	1	1.000	0.000	0.000	0.000	0.000	#N/A
	10366m5	30	6	3.930	1.518	0.833	0.746	0.758	−0.118
TUK	8346m3	23	3	1.301	0.446	0.174	0.232	0.237	0.249
	11401m4	23	7	3.963	1.591	0.783	0.748	0.764	−0.047
	8499m4	20	4	1.695	0.800	0.400	0.410	0.421	0.024
	Amil2-2	22	4	1.517	0.646	0.409	0.341	0.349	−0.200
	Amil2-23	23	1	1.000	0.000	0.000	0.000	0.000	#N/A
	Amil2-10	22	3	1.148	0.292	0.136	0.129	0.132	−0.056
	Amil2-12	22	1	1.000	0.000	0.000	0.000	0.000	#N/A
	10366m5	22	6	4.017	1.510	0.500	0.751	0.768	0.334
TOI	8346m3	25	4	1.228	0.420	0.200	0.186	0.189	−0.078
	11401m4	20	8	5.442	1.827	0.850	0.816	0.837	−0.041
	8499m4	25	6	1.611	0.850	0.360	0.379	0.387	0.051
	Amil2-2	22	5	1.847	0.884	0.364	0.459	0.469	0.207
	Amil2-23	25	3	1.176	0.324	0.160	0.150	0.153	−0.070
	Amil2-10	25	2	1.041	0.098	0.040	0.039	0.040	−0.020
	Amil2-12	25	1	1.000	0.000	0.000	0.000	0.000	#N/A
	10366m5	25	6	4.545	1.608	0.560	0.780	0.796	0.282
KAHyn	8346m3	24	3	1.135	0.274	0.125	0.119	0.121	−0.051
	11401m4	24	6	2.730	1.323	0.417	0.634	0.647	0.342
	8499m4	24	6	1.426	0.700	0.167	0.299	0.305	0.442
	Amil2-2	24	8	3.105	1.501	0.708	0.678	0.692	−0.045
	Amil2-23	24	2	1.280	0.377	0.250	0.219	0.223	−0.143
	Amil2-10	24	3	1.237	0.404	0.125	0.192	0.196	0.348
	Amil2-12	24	2	1.043	0.101	0.042	0.041	0.042	−0.021
	10366m5	24	7	3.789	1.514	0.708	0.736	0.752	0.038
KAHae	8346m3	14	2	1.074	0.154	0.071	0.069	0.071	−0.037
	11401m4	13	5	1.977	1.024	0.308	0.494	0.514	0.377
	8499m4	13	3	1.266	0.431	0.231	0.210	0.218	−0.099
	Amil2-2	14	5	3.698	1.443	0.643	0.730	0.757	0.119
	Amil2-23	14	2	1.690	0.598	0.429	0.408	0.423	−0.050
	Amil2-10	14	4	1.675	0.761	0.357	0.403	0.418	0.114
	Amil2-12	14	2	1.074	0.154	0.071	0.069	0.071	−0.037
	10366m5	14	7	6.031	1.852	0.500	0.834	0.865	0.401
OAH	8346m3	9	3	1.573	0.655	0.111	0.364	0.386	0.695
	11401m4	9	5	2.077	1.051	0.333	0.519	0.549	0.357
	8499m4	9	4	2.656	1.168	0.667	0.623	0.660	−0.069
	Amil2-2	9	3	2.314	0.937	0.444	0.568	0.601	0.217
	Amil2-23	9	2	1.528	0.530	0.222	0.346	0.366	0.357
	Amil2-10	9	3	1.588	0.684	0.444	0.370	0.392	−0.200
	Amil2-12	9	2	1.117	0.215	0.111	0.105	0.111	−0.059
	10366m5	9	5	3.375	1.353	0.556	0.704	0.745	0.211
MYK	8346m3	9	3	1.256	0.426	0.222	0.204	0.216	−0.091
	11401m4	9	6	3.600	1.504	0.444	0.722	0.765	0.385
	8499m4	9	4	2.282	1.040	0.556	0.562	0.595	0.011
	Amil2-2	9	2	1.670	0.591	0.556	0.401	0.425	−0.385
	Amil2-23	8	1	1.000	0.000	0.000	0.000	0.000	#N/A
	Amil2-10	9	4	1.800	0.855	0.556	0.444	0.471	−0.250
	Amil2-12	9	3	1.256	0.426	0.222	0.204	0.216	−0.091
	10366m5	9	5	4.378	1.519	0.778	0.772	0.817	−0.008

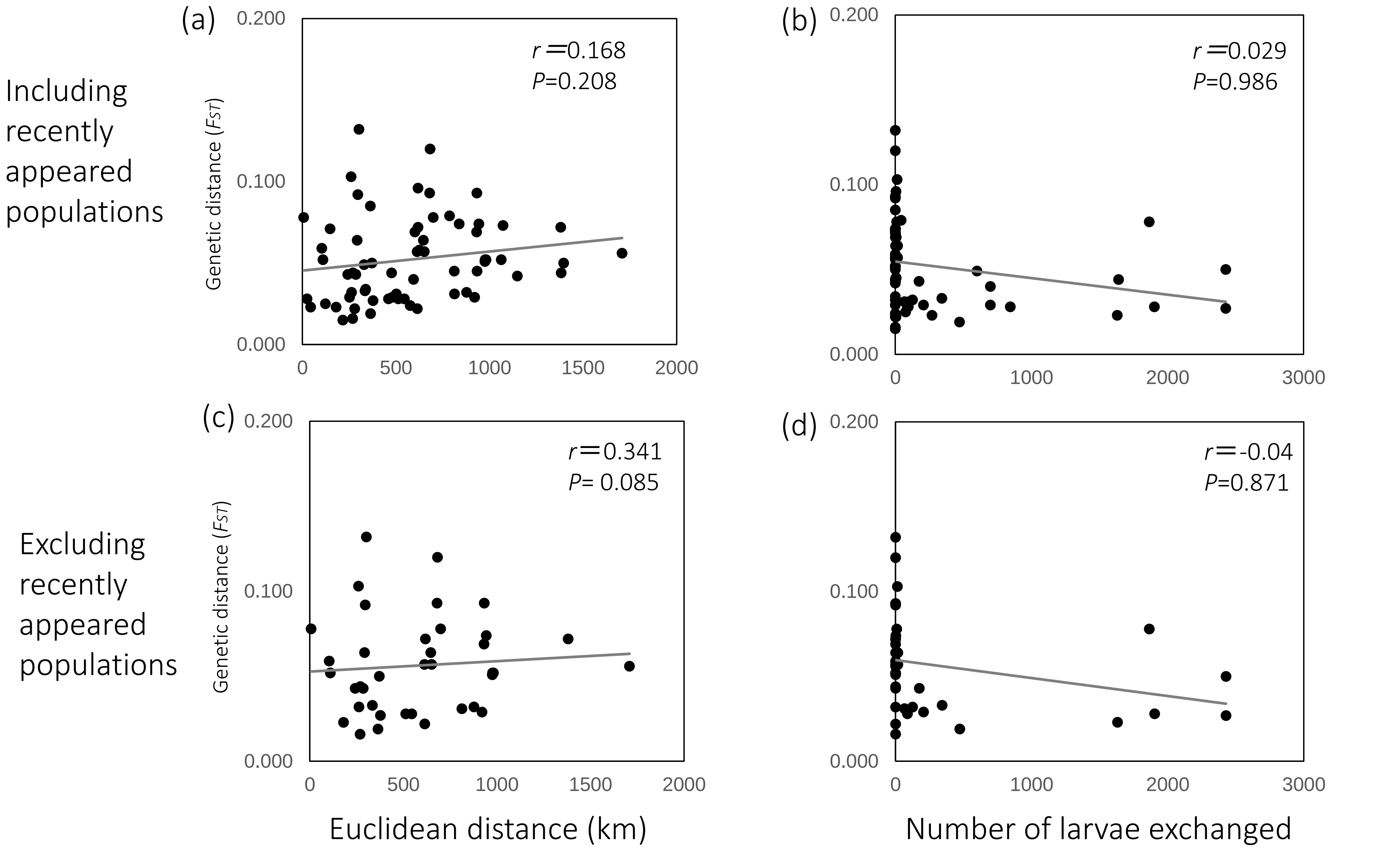

Suppl. 4 IBD patterns based on different geographic distances (a and c used Euclidean distance, b and d used number of larvae exchanged between sites based on numerical simulation) with different sample sets (a,b all populations, c,d excluding recently colonized populations).

(a)

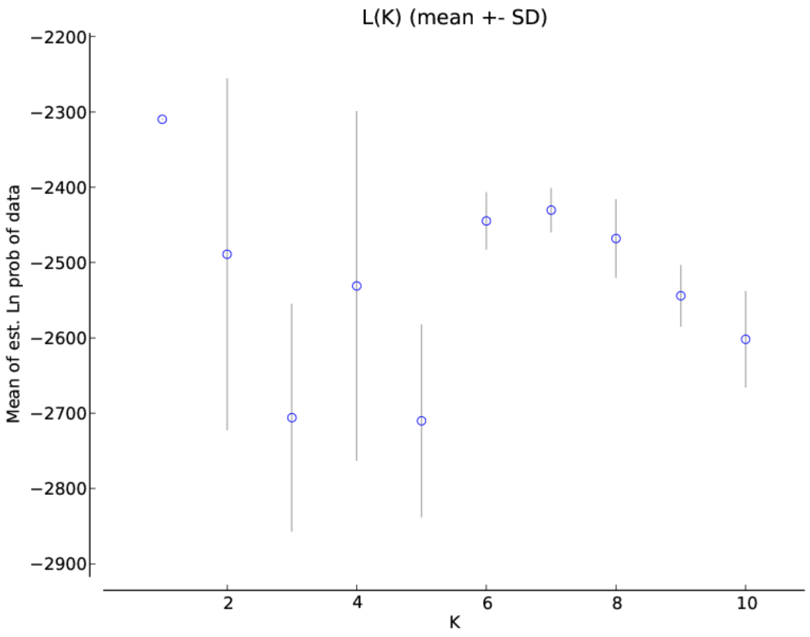

(b)

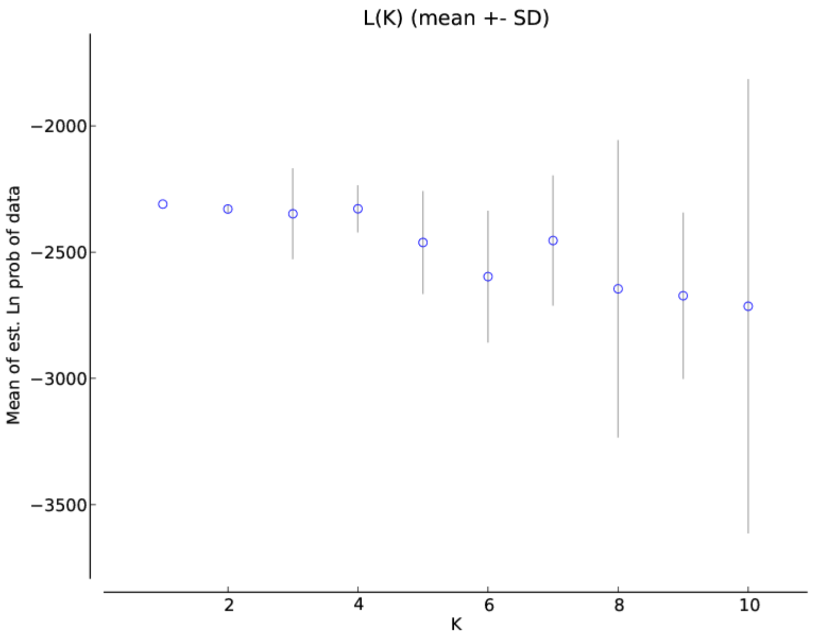

Suppl. 5 Likelihood values of STRUCTURE analysis only using green lineage (a) No prior (b) LOCPRIOR model used (temperate or subtropical regions).

Suppl. 6 Recent migration based on assignment test results using GeneClass2. Numbers indicate the number of individuals assigned to a population. Bold face indicates self-seeding. Columns are source populations, and rows are sink populations.

Assigned population / Source population		recently colonized in temperate			pre–existing in temperate					subtropical area		
		GAH	AMK	ZAH	WAH	KOC	TUK	TOI	KAHyn	KAHae	OAH	MYK
recently colonized in temperate area	GAH	**8**		1	2							
	AMK		**5**									
	ZAH			**2**								
pre–existing in temperate area	WAH	1			**13**							
	KOC					**5**						
	TUK				1		**21**	1				
	TOI							**6**				
	KAHyn								**9**			
subtropical area	KAHae					1				**9**		
	OAH									1	**3**	
	MYK											**6**
Unassigned		10	18	14	31	24	2	18	15	4	6	3

Suppl. 7 Environmental parameters of different regions. Data from NASA Goddard Space Flight Center, O. E. L., Ocean Biology Processing Group [25] and Yara, Y. et al. [26]

REGION	Water temperature average lowest month (℃)	Chlorophylla average (μl/L)	POC average (mg/㎥)	Aragonite saturation Ω −arag
Okinawa	21.98	0.17	54.24	3.30
Yakushima	19.56	0.26	76.30	3.10
Shikine	15.85	0.43	101.75	2.60
Amakusa−Goto	15.47	0.64	174.91	2.90

[25] NASA Goddard Space Flight Center, Ocean Ecology Laboratory, Ocean Biology Processing Group; (2018): MODIS-Aqua Level-2 Ocean Color Data Version, NASA OB.DAAC. http://doi.org/10.5067/AQUA/MODIS/L2/OC/2018. Accessed on 2018/01/01.
[26] Ocean acidification limits temperature-induced poleward expansion of coral habitats around Japan. Biogeosciences 9, 4955-4968 (2012).

Suppl. 8 Microsatellite markers used in this study.

Microsatellite locus	Primer F	Primer R	Repeat motif	Color	Multiplex	Reference
8346m3	CGACAAAGATTGGAGACCC	TTTCAATGCAGTGTGATTCC	(ATT)12	FAM (Blue)	Plex 1	Shinzato et al., 2014
11401m4	TGCAGACAGAACCGAGAAGG	TGGGCCACGATTCTTACG	(ATTT)8	NED (Yellow)		
8499m4	AAACCGTGGGTTAAGGGC	CGATGGAATTATTCGCGG	(CGGT)5	PET (Red)		
10366m5	CAACGACTGAAAGGCAGC	GGCTTTCGACTTTTATGTCC	(AAAAC)5	NED (Yellow)		
Amil2-2h	AATAACCCCTTCTACCTCTCT	TCTACAGCCGATTGTCAAGG	(CA)8	PET (Red)	Plex2	modified van Oppen et al., 2007
Amil2-23h	GTGTTACTGCATCAAACTG	GTGAGCATCCAAAGGTTTCC	(CT)5	NED (Yellow)		
Amil2-10	CAGCGATTAATATTTTAGAACAGTTTT	CGTATAAACAAATTCCATGGTCTG	TA(TG)11	FAM (Blue)		van Oppen et al., 2007
Amil2-12	TTTTAAAATGTGAAATGCATATGACA	TCACCTGGGTCCCATTTCT	GA(CA)6GA(CA)2	VIC (Green)		

Suppl. 10 Distances and the simulated larval dispersal exchange between pairs of populations

category	Pairwise compariso		Linearlized Fst	straightline distance	oceanog raphic_ distance	42d simulation 1 generation	42 simulation multiple generation	42 d simulation multiple generation_lg
recent	GAHo	GAHk	0.078	7.1	7.33	1704	1.9E+03	5.E-04
recent		AMK	0.059	108.04	110.31	1E-24	1.1E-01	9.E+00
recent		ZAH	0.052	976.9	1423.44	1E-24	6.8E-02	1.E+01
recent		WAHk	0.064	646.47	919.78	2	1.6E+01	6.E-02
recent		WAHs	0.057	612.77	897.77	1E-24	2.0E-01	5.E+00
recent		KOC	0.050	372.32	628.49	2231	2.4E+03	4.E-04
recent		TUK	0.102	264.21	436.04	415	5.1E+02	2.E-03
recent		TOI	0.032	265.16	386.92	1E-24	1.1E-01	9.E+00
recent		KAHyn	0.043	285.27	311.47	1E-24	5.2E-01	2.E+00
recent		KAHae	0.092	475.29	1428.37	1E-24	1.1E-02	9.E+01
recent		OAH	0.093	679.58	1161.03	1E-24	3.5E-05	3.E+04
recent		MYK	0.069	933.26	1034.22	1E-24	1.8E-07	6.E+06
recent	GAHk	AMK	0.052	114.25	105.09	1E-24	1.1E-01	9.E+00
recent		ZAH	0.052	981.76	1412.67	1E-24	6.8E-02	1.E+01
recent		WAHk	0.057	651.67	915.69	2	1.6E+01	6.E-02
recent		WAHs	0.072	617.75	894.58	1E-24	2.0E-01	5.E+00
recent		KOC	0.027	378	622.98	2231	2.4E+03	4.E-04
recent		TUK	0.169	271.27	432.45	415	5.1E+02	2.E-03
recent		TOI	0.044	272.25	381.67	1E-24	1.1E-01	9.E+00
recent		KAHyn	0.064	291.79	307.7	1E-24	5.2E-01	2.E+00
recent		KAHae	0.132	479.88	1423.95	1E-24	1.1E-02	9.E+01
recent		OAH	0.120	682.61	1145.58	1E-24	3.5E-05	3.E+04
recent		MYK	0.093	934.54	1034.91	1E-24	1.8E-07	6.E+06
recent	AMK	ZAH	0.032	874.16	1513.76	1E-24	1.3E+02	8.E-03
recent		WAHk	0.028	541.88	1011.11	23	8.8E+01	1.E-02
recent		WAHs	0.028	509.7	986.3	1173	1.9E+03	5.E-04
recent		KOC	0.016	265.49	720.82	1E-24	2.3E-02	4.E+01
recent		TUK	0.128	171.49	520.13	1E-24	2.1E-02	5.E+01
recent		TOI	0.023	176.31	472.99	505	1.6E+03	6.E-04
recent		KAHyn	0.043	237.13	406.35	28	1.7E+02	6.E-03
recent		KAHae	0.103	468.39	1515	11.33045623	1.3E+01	8.E-02
recent		OAH	0.078	697.01	1250.82	9.15434606	9.2E+00	1.E-01
recent		MYK	0.051	974.86	1126.35	1E-24	3.6E-02	3.E+01
recent	ZAH	WAHk	0.033	334.48	637.46	79	3.4E+02	3.E-03
recent		WAHs	0.019	364.45	709.68	94	4.7E+02	2.E-03
recent		KOC	0.022	613.22	875.59	1E-24	4.1E-02	2.E+01
recent		TUK	0.108	797.3	1034.32	1E-24	1.0E-02	1.E+02
recent		TOI	0.031	808.66	1050.94	1E-24	6.6E+01	2.E-02
recent		KAHyn	0.029	920.71	1158.41	51	2.1E+02	5.E-03
recent		KAHae	0.074	1135.32	1486	1E-24	1.9E+00	5.E-01
recent		OAH	0.072	1381.01	2248.71	1E-24	8.4E-03	1.E+02
recent		MYK	0.056	1705.74	2129.23	1E-24	2.8E-05	4.E+04

category	Pairwise compariso		Linearlized Fst	straightline distance	oceanog raphic_ distance	42d simulation 1 generation	42 simulation multiple generation	42 d simulation multiple generation_lg
Pre-exsited	WAHk	WAHs	0.023	44.18	62.64	201	2.7E+02	4.E-03
Pre-exsited		KOC	0.022	278.94	362.95	1	4.2E+00	2.E-01
Pre-exsited		TUK	0.118	465.35	524.88	2	3.7E+00	3.E-01
Pre-exsited		TOI	0.029	477.32	540.97	558	7.0E+02	1.E-03
Pre-exsited		KAHyn	0.040	593.77	646.79	558	7.0E+02	1.E-03
Pre-exsited		KAHae	0.096	822.23	985.68	1E-24	4.0E+00	3.E-01
Pre-exsited		OAH	0.073	1072.97	1751.5	1E-24	1.4E-02	7.E+01
Pre-exsited		MYK	0.050	1393.67	1623.42	1E-24	3.0E-02	3.E+01
Pre-exsited	WAHs	KOC	0.029	250.93	341.41	1E-24	4.9E-02	2.E+01
Pre-exsited		TUK	0.099	444.37	507.04	1E-24	3.9E-02	3.E+01
Pre-exsited		TOI	0.028	456.99	519.7	334	8.5E+02	1.E-03
Pre-exsited		KAHyn	0.024	576.63	629.14	1E-24	2.1E-01	5.E+00
Pre-exsited		KAHae	0.069	811.42	941.76	1E-24	5.7E+00	2.E-01
Pre-exsited		OAH	0.052	1063.25	1736.32	1E-24	2.5E-02	4.E+01
Pre-exsited		MYK	0.044	1381.69	1612.41	1E-24	3.0E-02	3.E+01
Pre-exsited	KOC	TUK	0.105	201.46	237.87	1429	1.6E+03	6.E-04
Pre-exsited		TOI	0.015	215.28	256.56	1E-24	2.5E-02	4.E+01
Pre-exsited		KAHyn	0.034	339.96	359.7	1E-24	5.4E-02	2.E+01
Pre-exsited		KAHae	0.085	588.32	683.25	1E-24	2.1E-03	5.E+02
Pre-exsited		OAH	0.074	840.14	1451.37	1E-24	7.2E-06	1.E+05
Pre-exsited		MYK	0.042	1150.26	1336.68	1E-24	5.1E-08	2.E+07
Pre-exsited	TUK	TOI	0.068	14.45	37.29	1E-24	2.0E-02	5.E+01
Pre-exsited		KAHyn	0.062	139.58	161.9	1E-24	5.4E-02	2.E+01
Pre-exsited		KAHae	0.087	392.08	533.97	1E-24	4.8E-04	2.E+03
Pre-exsited		OAH	0.087	642.05	1312.98	1E-24	1.6E-06	6.E+05
Pre-exsited		MYK	0.100	949.2	1183.07	1E-24	1.5E-08	7.E+07
Pre-exsited	TOI	KAHyn	0.025	125.29	120.22	1	7.4E+01	1.E-02
Pre-exsited		KAHae	0.071	377.63	499.53	1E-24	7.7E-01	1.E+00
Pre-exsited		OAH	0.058	627.67	1273.65	1E-24	4.3E-01	2.E+00
Pre-exsited		MYK	0.045	935.16	1151.99	1E-24	6.0E+00	2.E-01
Pre-exsited	KAHyn	KAHae	0.028	254.04	1264	72	9.4E+01	1.E-02
Pre-exsited		OAH	0.031	502.68	1006.51	53	1.0E+02	1.E-02
Pre-exsited		MYK	0.045	810.31	883.48	1E-24	3.5E+00	3.E-01
Pre-exsited	KAHae	OAH	0.044	252.47	533.87	1014	1.6E+03	6.E-04
Pre-exsited		MYK	0.079	571.72	752.09	1E-24	4.3E+01	2.E-02
Pre-exsited	OAH	MYK	0.049	326	487.42	67	6.0E+02	2.E-03
